# Can We Form Mesoporous Zeolites by Steam Assisted Crystallization of MCM-41?

**DOI:** 10.3390/molecules27248934

**Published:** 2022-12-15

**Authors:** Iane M. S. Souza, Sibele B. C. Pergher, Alexander Sachse

**Affiliations:** 1 Institut de Chimie des Milieux et Matériaux de Poitiers (IC2MP), Université de Poitiers, UMR 7285 CNRS, 4 rue Michel Brunet, CEDEX 9, 86073 Poitiers, France; 2 LABPEMOL, Laboratorio de Peneiras Moleculares. Av. Sen. Salgado Filho, 3000-Lagoa Nova, Natal 59072-970, RN, Brazil

**Keywords:** hierarchical zeolites, steam assisted crystallization, silicalite-1, MCM-41, MFI

## Abstract

The possibility of crystallizing silicalite-1 (MFI) from the pore walls of as-synthesized MCM-41 via steam-assisted crystallization (SAC) was thoroughly investigated. A kinetic study was conducted through the impregnation of as-synthesized MCM-41 with the structure-directing agent tetrapropyl-ammonium hydroxide (TPAOH). Materials obtained after different SAC treatment times (1–288 h) were characterized by XRD, nitrogen physisorption at 77 K, TGA/DTA, and SEM. The achieved results allowed us to conclude that during SAC treatment, rapid destruction of the hexagonal mesophase occurs with the enlargement of mesopores, probably by their coalescence, until achieving non-porous amorphous silica. Only thereafter is the crystallization of the MFI phase evidenced through the development of micron-sized (>10 µm) MFI structured crystals. This study suggests the probable practical impossibility of even partial crystallization of the pore walls of mesoporous materials by SAC.

## 1. Introduction

Zeolites are widely applied in several industrial processes, especially as catalysts and separation agents in oil refining and petrochemicals. This can be ascribed to their unique structural and chemical properties, including acidity and a high thermal and chemical stability, allowing for the induction of shape selectivity. However, due to the small pore size of the microporous system, generally below 1 nm, slow diffusion prevails. Moreover, as a result of this long diffusion path, severe diffusion limitations result, leading to low effectiveness in catalytic processes [1].

Mesoporous materials, such as the M41S family, were believed to overcome some of the drawbacks imposed by the micropores in zeolite catalysis [2]. However, their low hydrothermal stability and weak catalytic activity seriously limit their practical applications [3]. Moreover, one of the key features in zeolite catalysis and adsorption is their ability to induce shape selectivity, which is not possible when using mesoporous materials [4].

One alternative is the design of hierarchical zeolites that combine both properties of zeolites and mesoporous materials. In such materials, the diffusion path length is reduced and the accessibility of bulky molecules to active sites is increased. A variety of strategies have been developed that allow for the preparation of hierarchical zeolites which can be divided into top-down and bottom-up techniques [5,6,7,8,9,10,11]. 

The first approach usually refers to destructive strategies and is commonly achieved through demetalation, such as dealumination or desilication [12,13,14]. Unbiased treatments that allow for removing aluminum and silicon in the same proportion from zeolite frameworks were further described by Valtchev and colleagues [15]. They highlighted that the only hierarchization strategy currently industrially applied is dealumination, due to its cost-effectiveness and its ability to generate zeolites with higher hydrothermal stability [16]. Constructive approaches are based on strategies that allow for simultaneously generating the zeolitic phase and intracrystalline mesoporosity. Such approaches typically rely on the use of surfactant molecules that have an amphiphilic character and zeolite structure-directing properties [17,18] and are thus more cost-efficient. 

An intermediate strategy can be defined as the zeolitization of the amorphous walls of mesoporous materials [19]. Several reports have described the crystallization of the amorphous pore walls using steam-assisted crystallization (SAC). Zhou et al. reported the synthesis of a hierarchical micro/mesoporous aluminosilicate via SAC methodology using TUD-1 as a mesoporous precursor. The authors found that several factors influence the final structure, such as the humidity and crystallization time [20]. Li Chen et al. synthesized hierarchical MCM-41/MFI through the ion-exchanges made by MCM-41, with tetrapropylammonium bromide (TPABr) followed by SAC, resulting in the formation of intracrystalline mesoporous silicalite-1 [21]. Li et al. reported the synthesis of a hierarchical meso-/microporous aluminosilicate via SAC using soft meso- and micropore templates based on a micro emulsion for the creation of macroporosity, resulting in the formation of a network of interconnected pores on three length scales [22,23]. Furthermore, SAC, by the impregnation of tetrapropylammonium hydroxide (TPAOH) on Al-SBA-16, was reported to allow the synthesis of hierarchical micro-mesoporous ZSM-5 zeolites, where the role of TPAOH was identified as to act as a structure-directing agent and as a mineralizer allowing for the incorporation of aluminum into the MFI framework [24].

In order to understand the limits of the crystallization of the pore walls of mesoporous materials by SAC, we conducted an ex situ kinetic study that allowed us to study the time-resolved transformation of the as-synthetized MCM-41 into silicalite-1. The findings revealed that the SAC treatment leads to an important reorganization of the mesophase with progressive enlargement of mesoporosity prior to crystallization of the zeolite phase; the presence of intracrystalline mesoporosity could not be detected at any time during the transformation. 

## 2. Results and Discussion

**Figure 1:**
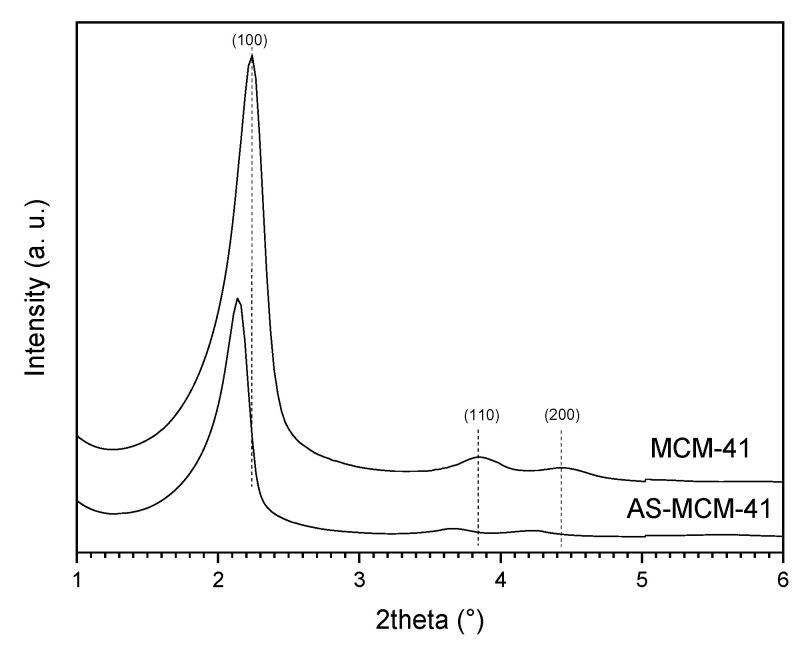
X-ray diffraction patterns for the as-synthetized MCM-41 (AS-MCM-41) and the calcined MCM-41 (MCM-41).

The transformation of as-synthetized MCM-41 (AS-MCM-41) was followed at different SAC treatment times after impregnation with TPAOH. The XRD patterns in the low angle range allow for observing the long-range order of the hexagonal mesophase array (Figure 1). The XRD patterns of the as-synthetized (AS-MCM-41) and the calcined mesoporous material (MCM-41) revealed three peaks corresponding to the ordered mesoporous MCM-41 and ascribable to the (100), (110), and (200) reflections typical for the hexagonal array [25]. By comparing the AS-MCM-41 and the calcined sample, a shift to higher 2θ values and an increase in the intensity of these peaks is observed, which results from the calcination and removal of the surfactant, with a reduction in the unit cell parameter (a_0_) from 4.77 to 4.55 nm for the AS-MCM-41 and the calcined MCM-41, respectively [26]. 

**Figure 2:**
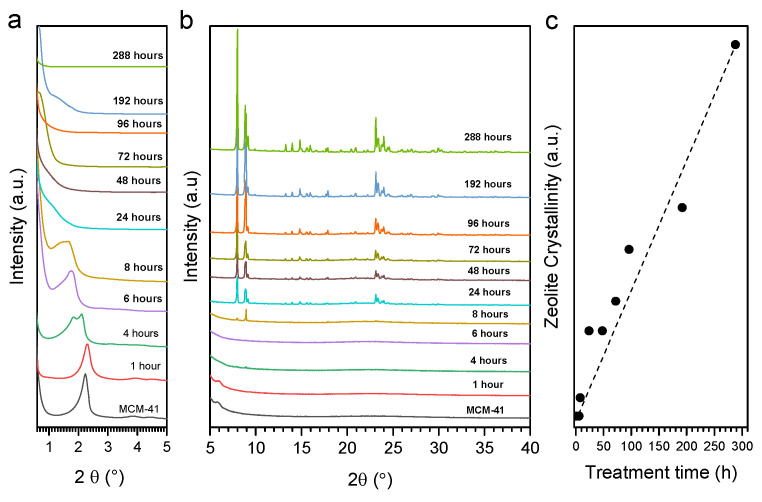
Low angle (**a**) and wide angle (**b**) X-ray diffraction patterns of materials after indicated times of SAC treatment and the evolution of zeolite crystallinity as a function of the SAC treatment time (**c**).

The loss of the long-range order of the mesophase within the first 24 h of the SAC treatment can be followed in Figure 2a. After 4 h of SAC, the (100) reflection becomes significantly wider and shifts to lower 2θ values, indicating a greater heterogeneity of mesopore size and a consolidation of the pore walls. XRD peaks associated with the MFI phase are first visible after 8 h of treatment through the appearance of reflections at 7.97 and 8.96° 2θ (Figure 2b). The reflections corresponding to the MFI phase are observable after 24 h of treatment. The zeolite crystallinity was estimated from the intensity of the XRD peaks at 7.97 and 8.96° 2θ (Figure 2c). A linear increase in the zeolite crystallinity could then be deduced. The XRD patterns hence indicate that only the sample after 8 h presents a somewhat ordered mesophase and the presence of zeolite crystallinity in low amounts (less than 5% in comparison with the crystallinity estimated for the sample after 288 h, for which full crystallinity into Silicalite-1 was assumed based on the micropore volume calculated by nitrogen physisorption). A similar observation was made for the crystal size estimated by the Scherrer equation (Figure S1).

**Figure 3:**
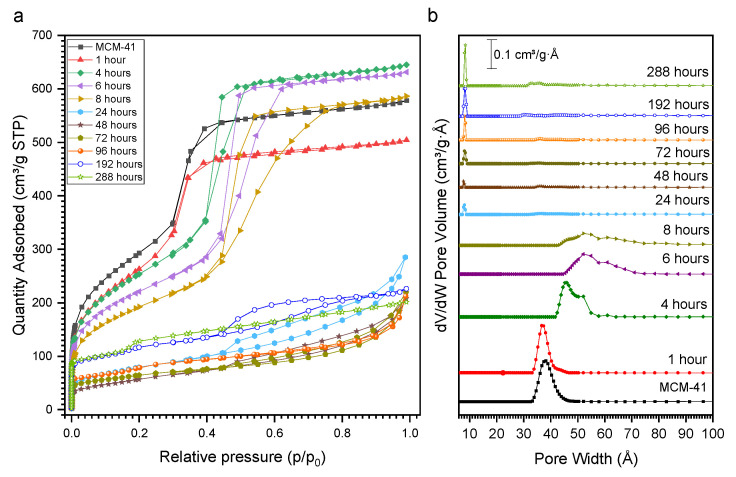
Nitrogen physisorption isotherms at 77 K (**a**) and DFT pore size distribution (**b**) for calcined materials achieved after different times of SAC treatment.

An important evolution of the shape of the nitrogen physisorption isotherms can be observed as a function of the SAC treatment time (Figure 3a). The parent MCM-41 presents a type IV(a) isotherm [27]. A very similar shape is observed for samples recovered within the first 1 h of SAC, with a slight reduction in the porous volume. Thereafter, the extent of the hysteresis loop increases steadily and the enlargement of the mesopores is observed (Figure 3b). The increase in the pore size distribution clearly indicates the coalescence of mesopores during SAC, as already indicated by the low-angle XRD patterns. After 24 h of SAC, the shape of the isotherms modifies significantly, featuring an H4 hysteresis loop and presenting a more pronounced nitrogen uptake in the low p/p_0_ region, indicating the presence of micropores. Indeed, in the DFT pore size distribution, a contribution centered at 8 Å can be distinguished (Figure 3b). After 192 h of SAC, the isotherms present a hysteresis that may be related to the presence of mesoporosity, however, the pore size distribution shows that at this time of transformation there is no more presence of porosity related to MCM-41 channels; in addition, the XRD results confirm a high crystallinity with no evidence of reflections associated with MCM-41. It is interesting to note that microporosity starts evolving once mesoporosity is destroyed, suggesting that the degradation of the mesophase first leads to the formation of a non-porous solid prior to the zeolite crystallization. The textural data for each sample are shown in Table S1.

**Figure 4:**
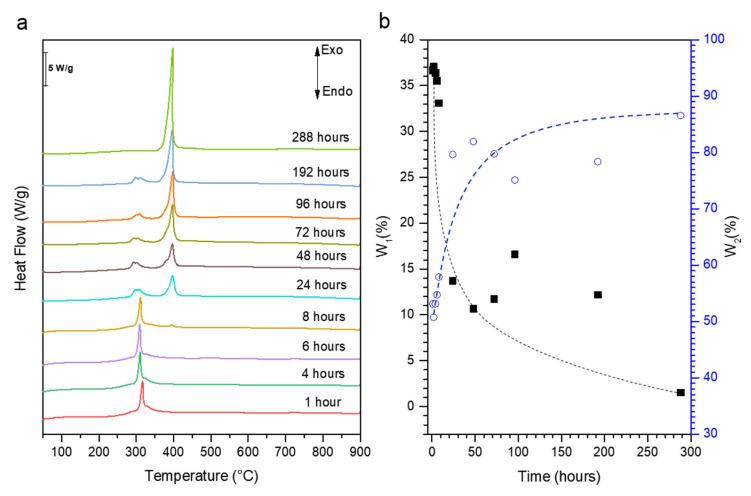
Differential thermogravimetric analysis (DTA) (**a**) and weight loss (**b**) for materials after indicated SAC times. The blue and black symbols represent the cumulative weight loss between 120 and 350 °C (W1) and 350 and 900 °C (W2).

The TGA of AS-MCM-41 presents a weight loss of 55% (Figure S2). The decomposition and combustion of cetyltrimethylammonium (CTA^+^) are observed starting from 150 °C and maximal heat flow is observed at 320 °C (Figure 4a) [28]. The weight loss associated with the decomposition and combustion of CTA^+^ reduces with increasing SAC treatment time (Figure 4b). This indicates the removal of the surfactant molecule from the mesopores to the outer surface where it is removed by washing with water after SAC treatment. After 8 h of SAC, a mass loss at a higher temperature (390 °C) can initially be observed, indicating the presence of TPA^+^ confined in zeolitic micropores. A slight weight loss contribution due to CTA^+^ degradation can be observed even after full crystallization into MFI, which is probably due to the CTA^+^ adsorption on the outer surface of the zeolite crystals. 

**Figure 5:**
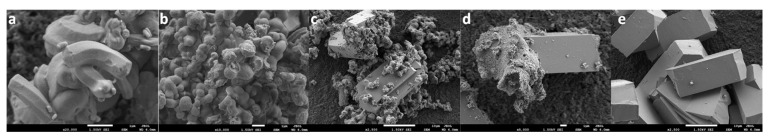
SEM images of parent MCM-41 ((**a**), length of the scale bars of 1 µm) and for materials achieved after SAC treatment for 8 h ((**b**), length of the scale bars of 1 µm), 24 h ((**c**), length of the scale bars of 10 µm), 96 h ((**d**), length of the scale bars of 1 µm), and 288 h ((**e**), length of the scale bars of 10 µm).

The evolution of the morphology was further followed by SEM. The SEM images of the parent MCM-41 present typically elongated rods of 2 µm length and 500 nm diameter (Figure 5a). After 8 h of SAC, the deformation of rods into spherical particles below 1 µm in size can be observed (Figure 4b). Upon 24 h of SAC, crystals of 20 µm length can first be seen, which present the typical shape of the MFI phase (Figure 5c). These are surrounded by a phase of undefined morphology, typical for amorphous materials. By further increasing the SAC treatment time, the number of observable crystals increases; after 288 h, the mere presence of the MFI crystals is observed (Figure 5e). 

## 3. Materials and Methods

Hexadecyltrimethylammonium bromide (CTAB, ≥98%), sodium hydroxide (NaOH, pellets, ≥98%), tetraethylorthosilicate (TEOS, 98%), and the tetrapropylammonium hydroxide (TPAOH, 40% solution in water) were purchased from Sigma-Aldrich.

The MCM-41 was synthesized following an adapted synthesis from [29], wherein 5 g of CTAB was dissolved in 450 mL of distilled water and 17.5 mL of NaOH 2M was added and stirred for 30 min. After that, 25 mL of TEOS was added and stirred for 2 h at 80 °C. The formed solid was centrifuged, washed until pH = 7, and dried at 80 °C overnight. The as-synthetized MCM-41 achieved was named AS-MCM-41. To obtain MCM-41, the AS-MCM-41 was calcined at 550 °C for 6 h with a heating ramp of 2 °C min^−1^ under air.

Steam-assisted crystallization (SAC) was carried out using 0.5 g of AS-MCM-41. A total of 0.2 mL of TPAOH 40% was dissolved in 4 mL of absolute ethanol and mixed with the AS-MCM-41 and stirred for 30 min. Thereafter, the liquid phase was evaporated at 80 °C. The dry solid was placed in a porcelain crucible and positioned inside a Teflon-lined autoclave containing 5 mL of distilled water outside the porcelain crucible and heated at 180 °C under autogenous pressure for 1 to 288 h. After the indicated time, the solid was collected, washed with distilled water, and centrifuged. The solid was then calcined at 550 °C for 6 h. 

The X-ray diffraction (XRD) patterns were obtained on an Empyrean PAN analytical with Cu-Kα emission (λ = 1.54 Å) in the range 2θ 0.5–5° and 2θ 5–50°. The zeolite crystallinity was estimated considering the intensity of the XRD peaks of the reflections (001) and (200). The zeolite crystal size was estimated by the Scherrer equation:

$$\beta_{S}{\left(2\theta \right)}_{hkl}=\frac{K\lambda }{Tcos\theta_{hkl}}$$

where *β_s_* is the full width at half maximum (FWHM), *K* is a constant near unity associated with the FWHM, and *T* is the average thickness of the crystal in a direction normal to the diffracting plane *hkl*. For the crystal size estimation, the (001), (200), (051), (431), and (242) reflections of the MFI phase were considered. The MCM-41 cell parameter was calculated from the equation in [30]:

$$a_{0}=2d_{100}/\sqrt{3}$$

Nitrogen physisorption isotherms at 77 K were measured on a Micromeritics TRIFLEX instrument. The pore size distribution was calculated by DFT using a cylindrical pore model. Micropore volume was calculated using a t-plot [31]. Thermogravimetric/differential thermal analysis (TGA/DTA) was carried out on an SDT Q600 TA 25 to 900 °C with a temperature ramp of 5 °C min^−1^ under a gas flow of 100 mL min^−1^. Scanning electron microscopy (SEM) was then performed using a JEOL JSM-790CF instrument.

## 4. Conclusions

The SAC treatment of as-synthetized MCM-41 with TPAOH leads to the very rapid degradation of the structured mesophase and its eventual loss. Complete mesophase degradation is first observed within 24 h of SAC, showing a significant zeolite crystallization thereafter. The achieved results thus indicate that the development of zeolites featuring intracrystalline mesoporosity is practically impossible by the SAC treatment of MCM-41 in the applied conditions due to the fast degradation kinetics of the mesosphase and the rather slow zeolite crystallization. The achieved results further indicate that SAC treatment on other mesoporous materials, such as SBA-15, could lead to similar results, given the very similar reactivity of the silica in such materials compared to MCM-41. The development of zeolite fragments on the coalescing mesopore walls cannot, however, be ruled out and will be the topic of further research. 

## Data Availability

Not applicable.

## Supplementary Materials

The following supporting information can be downloaded at: https://www.mdpi.com/article/10.3390/molecules27248934/s1, Figure S1. Evolution of crystal size estimated by the Scherrer equation as a function of SAC time and Figure S2. Thermogravimetric analysis under synthetic air for samples achieved after different SAC treatment times. Table S1: Textural properties of parent calcined MCM-41 and of materials after different SAC treatment times.

## Sample Availability

Samples of the compounds are available from the authors.
