# Supplementary material for: Can We Form Mesoporous Zeolites by Steam Assisted Crystallization of MCM-41?
Source: Molecules. 2022 Dec 15;27(24):8934. doi: 10.3390/molecules27248934 (PMC9783799; doi:10.3390/molecules27248934)
Supplement: Supplementary file 1 [file molecules-27-08934-s001.zip › molecules-2055400-supplementary.pdf]

Supplementary Information

Can we form mesoporous zeolites by steam assisted crystallization of MCM-41?

Iane M. S. Souza [1,2], Sibele B. C. Pergher [2] and Alexander Sachse [1,*]

[1] Institut de Chimie des Milieux et Matériaux de Poitiers (IC2MP), Université de Poitiers, UMR 7285 CNRS, 4 rue Michel Brunet, CEDEX 9, 86073 Poitiers, France
[2] LABPEMOL, Laboratorio de Peneiras Moleculares. Av. Sen. Salgado Filho, 3000-Lagoa Nova, Natal 59072-970, RN, Brazil
* Correspondence: alexander.sachse@univ-poitiers.fr

Table S1. Textural properties of parent calcined MCM-41 and of materials after different SAC treatment times.

Sample	S_{BET} (m^2/g)	S_{micro} (m^2/g)	t-plot external surface (m^2/g)	V_{TP} (cm^3/g)	V_{μ}(cm^3/g)	Pore width (Å)
MCM-41	1090	0	1000	0.90	0	38
1 hour	980	0	965	0.78	0	37
4 hours	930	0	964	0.99	0	45
6 hours	800	0	750	0.97	0	53
8 hours	700	0	650	0.90	0	53
24 hours	290	45	237	0.45	0.02	8
48 hours	215	70	140	0.31	0.03	8
72 hours	225	115	110	0.34	0.05	8
96 hours	290	185	105	0.31	0.07	8
192 hours	420	250	170	0.35	0.09	8
288 hours	440	345	95	0.31	0.12	8

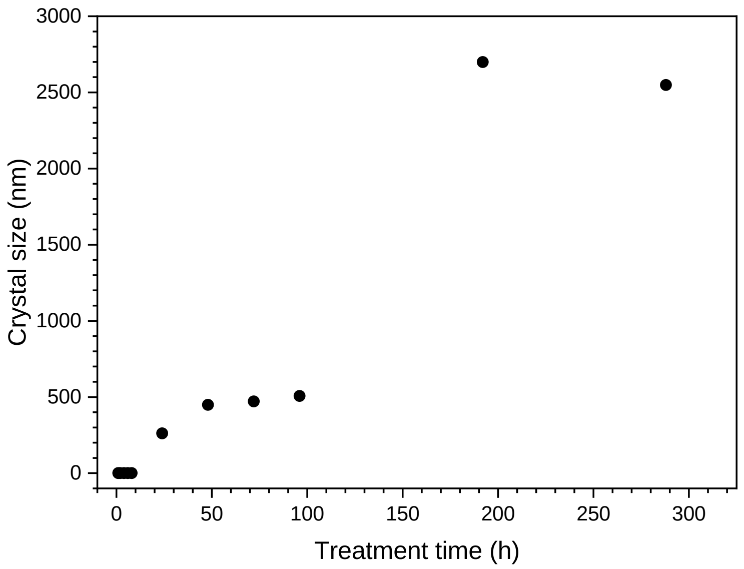

Figure S1. Evolution of the crystal size estimated by the Scherrer equation as a function of the SAC treatment time.

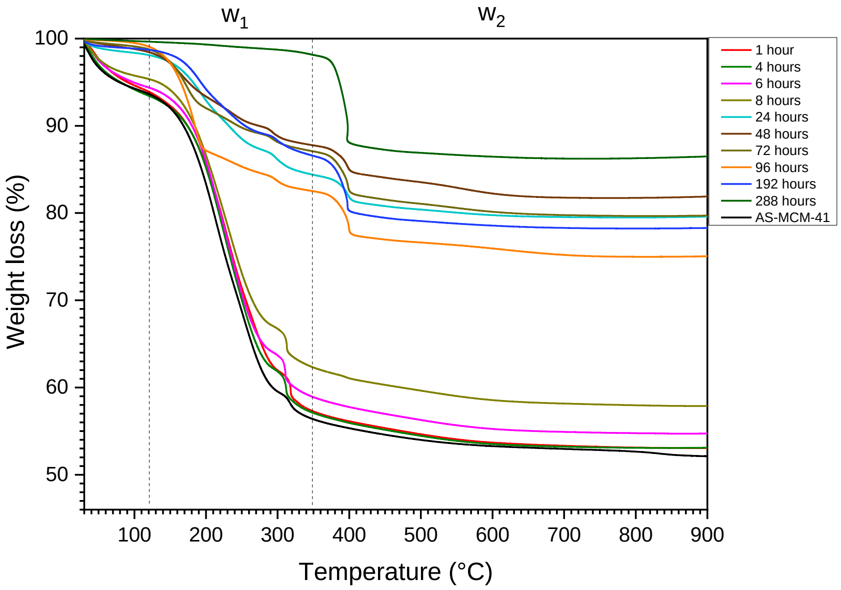

Figure S2. Thermogravimetric analysis under synthetic air for samples achieved after different SAC treatment times.
